# The posttraumatic stress disorder project in Brazil: neuropsychological, structural and molecular neuroimaging studies in victims of urban violence

**DOI:** 10.1186/1471-244X-9-30

**Published:** 2009-06-01

**Authors:** Rodrigo A Bressan, Lucas C Quarantini, Sérgio B Andreoli, Celia Araújo, Gerome Breen, Camila Guindalini, Marcelo Hoexter, Andrea P Jackowski, Miguel R Jorge, Acioly LT Lacerda, Diogo R Lara, Stella Malta, Tais S Moriyama, Maria I Quintana, Wagner S Ribeiro, Juliana Ruiz, Aline F Schoedl, Ming C Shih, Ivan Figueira, Karestan C Koenen, Marcelo F Mello, Jair J Mari

**Affiliations:** 1 Laboratório Interdisciplinar de Neurosciencias Clínicas – LiNC, São Paulo, Brazil; 2 Department of Psychiatry, Universidade Federal de São Paulo, Brazil; 3 Instituto Israelita de Ensino e Pesquisa Albert Einstein, São Paulo, Brazil; 4 Department of Society, Human Development, and Health, Harvard School of Public Health, Cambridge, MA, USA; 5 Depart of Psychiatry, Universidade Federal da Bahia, Bahia, Brazil; 6 MRC Social, Genetic and Developmental Psychiatry Research Centre and NIHR Biomedical Research Centre for Mental Health at NHS South London, UK; 7 Maudsley NHS Foundation Trust and Institute of Psychiatry, King's College London, UK; 8 Faculdade de Biociências da PUCRS, Brazil; 9 Institute of Psychiatry, Universidade Federal do Rio de Janeiro (IPUB – UFRJ), Rio de Janeiro, Brazil; 10 Centre for Public Mental Health, Health Services and Population Research Department, Institute of Psychiatry, King's College, University of London, London, UK

## Abstract

**Background:**

Life trauma is highly prevalent in the general population and posttraumatic stress disorder is among the most prevalent psychiatric consequences of trauma exposure. Brazil has a unique environment to conduct translational research about psychological trauma and posttraumatic stress disorder, since urban violence became a Brazilian phenomenon, being particularly related to the rapid population growth of its cities. This research involves three case-control studies: a neuropsychological, a structural neuroimaging and a molecular neuroimaging study, each focusing on different objectives but providing complementary information. First, it aims to examine cognitive functioning of PTSD subjects and its relationships with symptomatology. The second objective is to evaluate neurostructural integrity of orbitofrontal cortex and hippocampus in PTSD subjects. The third aim is to evaluate if patients with PTSD have decreased dopamine transporter density in the basal ganglia as compared to resilient controls subjects. This paper shows the research rationale and design for these three case-control studies.

**Methods and design:**

Cases and controls will be identified through an epidemiologic survey conducted in the city of São Paulo. Subjects exposed to traumatic life experiences resulting in posttraumatic stress disorder (cases) will be compared to resilient victims of traumatic life experiences without PTSD (controls) aiming to identify biological variables that might protect or predispose to PTSD. In the neuropsychological case-control study, 100 patients with PTSD, will be compared with 100 victims of trauma without posttraumatic stress disorder, age- and sex-matched controls. Similarly, 50 cases and 50 controls will be enrolled for the structural study and 25 cases and 25 controls in the functional neuroimaging study. All individuals from the three studies will complete psychometrics and a structured clinical interview (the Structured Clinical Interview for DSM-IV and the Clinician-Administered PTSD Scale, Beck Anxiety Inventory, Beck Depression Inventory, Global Assessment of Function, The Social Adjustment Scale, Medical Outcomes Study 36-Item Short-Form Health Survey, Early Trauma Inventory, Clinical global Impressions, and Peritraumatic Dissociative Experiences Questionnaire). A broad neuropsychological battery will be administered for all participants of the neuropsychological study. Magnetic resonance scans will be performed to acquire structural neuroimaging data. Single photon emission computerized tomography with [(99m)Tc]-TRODAT-1 brain scans will be performed to evaluate dopamine transporters.

**Discussion:**

This study protocol will be informative for researchers and clinicians interested in considering, designing and/or conducting translational research in the field of trauma and posttraumatic stress disorder.

## Background

Posttraumatic stress disorder (PTSD) occurs following exposure to a potentially traumatic life event and is defined by three symptom clusters: reexperiencing, avoidance and numbing, and arousal [1]. However, PTSD is relatively rare event in trauma-exposed people. This fact has motivated research aimed at identifying risk factors for this disorder. Two meta-analyses of PTSD risk factors have come to some consensus as to the key factors influencing PTSD vulnerability. These include small but consistent effects on risk for pre-trauma factors such as cognitive ability, family psychiatric history, pre-trauma psychological adjustment, child abuse, other previous trauma exposures, and general childhood adversity [2,3]. Characteristics of the traumatic experience were found to be particularly important, especially trauma severity, perceived life threat and peri-traumatic emotional reactions such as dissociation [2,3]. A dose-response relation between severity of exposure and conditional risk of developing PTSD has been well-documented [4,5]. Post-trauma social support also appears to play a role [2,3]. However, the risk factors models supported by meta-analytic studies explain only about 20% of the variance in PTSD; clearly new variables need to be incorporated into models of PTSD vulnerability. Due in part to methodological limitations of extant research, the role of neuropsychological and brain structure and functional factors in the etiology of PTSD are less well understood.

This paper describes the protocol for the Project Post-Traumatic Stress Disorder in Brazil which is aimed at characterizing the underlying biology of PTSD neuropsychological assessment, neurostructural evaluation and molecular imaging of the dopamine transporter system. Brazil offers a unique environment to conduct translational research about psychological trauma and PTSD. From 1980 to 2000, a total of more than 598 thousand people died in Brazil because of homicide with two thirds of this occurring in the 90's. In 1980 the leading cause of violent death in the country was traffic accidents but in 2000 it was homicides [6,7]. From 1991 to 2000 there was an increase of 27% in the proportion of deaths caused by homicides among the total deaths in the country. Thus, lethal violence became a national phenomenon, being particularly related to the rapid population growth of large and urban cities [8].

### Neuropsychological findings in PTSD

PTSD is characterized by re-experiencing of the traumatic event and the inability to consciously recall facts about the traumatic event as well as by altered emotional processing of trauma-relevant cues. The patient's memory seems to be fixed on the traumatic event, and the retrieval of others memories seems to be inhibited [9-11]. Previous research on the neuropsychology of PTSD has identified several neurocognitive deficits [12]. Neuropsychological measures of intellectual ability, learning, memory (verbal and non-verbal), attention, visuospatial ability, executive functioning, language, psychomotor speed have been examined. Several studies have identified impaired performance on verbal memory and learning in PTSD cases as compared to controls [13-16]. Other studies have documented differences between cases and controls in different domains, including attention, working memory [17-22], and processing speed [21]. Although there is evidence for memory impairment in PTSD subjects, it remains unclear whether memory impairment is confined to verbal material or nonverbal material is also affected [11,14,21]. Different studies, have found impaired visual memory in individuals with PTSD [18,19,23,24].

Many studies have shown impairments in the whole mnemonic process (immediate memory, recall and recovery), attention, learning, intellectual level, and emotional processing [25]. Nonetheless, others studies have shown no differences between cases and controls for memory [24,26-29] and/or attention performance [12,13,30]. Those neuropsychological findings suggest involvement of two primary strcutures: hippocampus and prefrontal cortex. Several studies have reported decreases in hippocampal volume [31-36] and hippocampal N-acetylaspartate [37-39] as well as an association between hippocampal atrophy and poor verbal memory in PTSD subjects [23]. Neurofunctional studies have indicated specific findings in limbic regions, although the relationship of these results to neuropsychological performance remains to be explored [40].

An alternative model of PTSD may be related to a dysfunction of higher-level attentional resource which in turn might affect activity in other systems concerned with memory and thought [20,41,42]. Attention and concentration difficulties appear to be core deficits in PTSD and memory deficits might actually be secondary to an impaired attention. A possible explanation for the association between memory and attention difficulties in PTSD is that explicit memory performance may be impacted by impaired attention resources during processing. Some researchers have theorized that the heightened emotional reactivity in patients with PTSD disrupts attentional resources [43]. Impaired attention prevents sufficient registration of information, which in turn prevents consolidation and retrieval of memory. Furthermore, recent studies have demonstrated a possible deficit in the inhibitory processes of memory in PTSD [18,19,44].

Several potential confounding factors must be considered for neuropsychological evaluation of PTSD patients including comorbid depression, substance abuse, and medical conditions, type of trauma, and motivational aspects. Therefore, any conclusion if deficits observed are specifically related to PTSD should take into account alternative explanations such as the possible effect of confounding factors [26,40]. In conclusion, neuropsychological functioning has emerged as promising endophenotype to be explored in PTSD.

### Neurostructural findings in PTSD

One of the possible mechanisms underlying the physiopathology of PTSD is the damaging action of glucocorticoids on hippocampus [45]. The hippocampus plays a central role in neuropsychological functions such as memory and emotional behaviour and is likely to be involved in the development of PTSD. An alternative model would be a failure in the regulatory activity of prefrontal cortex over amygdala, with a consequent hyperactivity of the later in response to traumatic memories [46-48]. This inhibitory deficiency is also a possible explanation for the hyperexcitability symptoms found in PTSD. The orbitofrontal cortex, the ventral portion of prefrontal cortex, appears to be central in the regulation of prefrontal cortex over amygdala and is also implicated in the processing of negative emotion [49]. Studies using different techniques have consistently found neurofunctional abnormalities in this region in PTSD patients [50-52]. Furthermore, some symptoms that may be seen in PTSD, such as poor impulse control and violent assaults, are also reported in individuals with lesions in orbitofrontal cortex [53,54]. Findings from magnetic resonance imaging studies have suggested neurostructural alterations in PTSD. Although these findings are less consistent than those seen in neurofunctional studies, volumetric reductions in hippocampus [23,32,34-36,51,52] and prefrontal regions [55,56] have been reliably found. However, additional studies are still warranted in order to assess whether these structural abnormalities are specific to PTSD or represent non-specific morphological abnormalities associated to trauma exposure. Further examination of abnormalities in frontolimbic structures are also required to clarify the role of structural and functional brain abnormalities in the pathophysiology of PTSD.

### Molecular imaging of dopamine transporter in PTSD

Physiological response to stressful experience involves a vast neural-endocrine-immunologic reaction that leads to the release of catecholamines and autonomic nervous system stimulation [57]. Noradrenergic and hypothalamus-pituitary-adrenal axis systems are the most common studied in response to stress [58] but many other neural-chemical systems are implicated. In animal studies, dopaminergic innervation of the basolateral nucleus of the amygdala, the medial prefrontal cortex and other limbic regions is highly responsive to stress and may be altered by stress [59-61]. Also the enhancement of the acoustic startle response, which can be a symptom of PTSD, has been related to the dopamine D1 receptor agonists in rats [62]. Genetically determined alterations in dopamine release and dopamine receptor expression in mice have been implicated in behavioral abnormalities induced by chronic stress [63]. This finding was interpreted as suggesting that stress-induced alterations of central dopaminergic neurotransmission may be genotype-dependent and expressed in behaviour. Human studies showed that there was a relationship between urinary excretion of dopamine and plasma dopamine and (the severity of) PTSD symptoms [64].

Evidence from genetic studies has proposed that reduced density of D2 dopaminergic receptor predisposes to PTSD [65]. There are two important PTSD candidate genes that directly affect the dopamine system: the dopamine receptor gene (DRD2) and the dopamine transporter (DAT) gene. The D2 dopamine receptor (DRD2) minor (A1) allele DRD2 A1 has already been linked to ADHD, Tourette's syndrome, conduct disorder and substance abuse [66]. This prompted suppositions that this gene may be involved in stress response in humans [61]. Polymorphism of the dopamine transporter (DAT) gene, in the locus SLC6A3 3' (VNTR), has been found to predispose to PTSD and to chronic forms of the disorder [67]. Taken together, these evidences suggest a relevant role for dopamine in the pathogenesis of PTSD.

To gain more clarity about any link between PTSD and the DAT, it is therefore important to clearly document PTSD patients, controlling confounders as alcohol consumption, major depression and clinical illness, through a functional neuroimaging investigation.

Although molecular imaging allows reliable information on in vivo dopaminergic function [68], no studies, to our knowledge, has examined dopaminergic system activity in PTSD patients using molecular neuroimaging techniques.

The main reasons that justify such an effort to understand the problem of violence and its consequences for mental health can be described as follows: 1) Treatment strategies to be sponsored by the Brazilian public health care system need to be based on solid local data on the extent and nature of the disorder; 2) Exposure to traumatic life events is related to not only with mental disorders which include PTSD and depression, but also with other cognitive and neurotransmission dysfunctions [18,19,44,69]. The impact in mental health of the population of exposure to violence among the population of Sao Paulo, a large urban centre in a Middle Income Country (MIC), as well as specific parameters such as neurocognitive, neurostructural anf functional neuroimaging finds is virtually unknown.

The subjects of the study will be selected from an epidemiological/genetic survey in the city of Sao Paulo to assess the relationship between exposure to violence and the prevalence of PTSD and common mental disorders. Subjects located in the epidemiological/genetic survey will be referred to these three case-control studies, reported here, and to a randomized controlled clinical trial on the efficacy of topiramate for the treatment of PTSD symptoms.

This protocol is the result of collaborative task force to conduct translational research in the field of traumatic stress in urban regions of Brazil. The current project investigates possible causes for neurocognitive deficits, neurostructural changes and dopaminergic dysfunction in individuals with PTSD. We will be comparing individuals with current or lifetime diagnosis of PTSD with those who were exposed to a traumatic event but did not develop a current or a lifetime diagnosis of PTSD.

The main objectives of this project are:

1. to examine cognitive functioning of PTSD subjects and its relationships with symptomatology;

2. to evaluate neurostructural integrity of orbitofrontal cortex and hippocampus in PTSD subjects;

3. to evaluate if patients with PTSD have decreased dopamine transporter density in the basal ganglia as compared to resilient controls subjects.

## Methods and design

### Sample

Cases and controls will be identified through an epidemiologic survey conducted in the city of São Paulo. Details of the epidemiologic study are presented in a companion paper (cite) and are summarized here. To identify trauma victims in the community, interviews were conducted by a professional team specialized in household surveys, the Brazilian Institute of Public Opinion and Statistics. Interviewers were trained at the CIDI [70] in the Federal University of São Paulo, an accredited center by the World Health Organization (WHO). Training procedures were conducted in accordance to the guidelines set up by the WHO. Interviews were carried out in the participants households by means of printed questionnaires. All questionnaires were translated into Portuguese and adapted to the local social and cultural context. Inclusion and exclusion criteria for the three case-control studies are described in tables 1 and 2, respectively. Individuals who met inclusion criteria during the epidemiologic study were invited to participate in the case-control study. Subjects exposed to traumatic life experiences resulting in PTSD (cases) will be compared to resilient subjects victims of traumatic life experiences without PTSD (controls) aiming to identify biological variables that might protect or predispose to PTSD. This case-control design will enrol representative subjects from the community being this procedure an important technique to overcome Berkson bias. Subjects will be informed about the procedures of the studies and will be asked to formally consent willingness to participate. Subjects who consent to participate in the case-control studies will receive the following assessment:

**Table 1:** Inclusion criteria for PTSD cases (p) and control (c) groups

	Neuropsychological Study		Structural Neuroimaging study		Molecular Neuroimaging study	
	p	C	P	c	p	c
Age between 18 and 60 (inclusive)	X	X	X	X	X	X
Life time history of traumatic life experience as defined in criteria A of DSM IV criteria for PTSD	X	X	X	X	X	X
PTSD diagnosis according DSM IV criteria as assessed by SCID I applied by trained psychiatrists or psychologists	X		X		X	
Good general health with no additional diseases expected to interfere with the study	X	X	X	X	X	X
Able to understand and signed informed consent	X	X	X	X	X	X
Completed 5 years grades of education	X	X				
						
Fluent in Portuguese	X	X	X	X	X	X
Willing and able to complete all assessments	X	X	X	X	X	X
Willing to undergo neuroimaging (MRI)			X	X		
Willing to undergo neuroimaging (SPECT)					X	X

**Table 2:** Exclusion criteria for PTSD cases (e) and control (c) groups

	Neuropsychological study		Structural Neuroimaging study		Molecular Neuroimaging study	
	
	p	c	p	c	P	c
Any significant neurologic disease, such as Parkinson's disease, multi-infarct dementia, Huntington's disease, normal pressure hydrocephalus, brain tumor, progressive supranuclear palsy, seizure disorder, subdural hematoma, multiple sclerosis, or history of significant head trauma followed by persistent neurologic defaults or known structural brain abnormalities.	X	X	X	X	X	X
Any significant systemic illness or unstable medical condition	X	X	X	X	X	X
History of significant head trauma followed by loss of consciousness			X	X		
Presence of pacemakers, aneurysm clips, artificial heart valves, ear implants, metal fragments or foreign objects in the eyes, skin or body.			X	X		
Claustrophobia			X	X		
Current use of psychoactive medications such as antidepressants, neuroleptics, anxiolytics or sedative hypnotics and mood stabilizers.			X	X	X	X
History of the following psychiatric disorders: schizophrenia, schizoaffective disorder, delusional disorder, bipolar affective disorder and depressive disorder with psychotic features (DSM IV criteria)	X	X	X	X	X	X
Tremor or dystonia in the cephalic region that unable the scanning procedure for imaging acquisition			X	X	X	X

### Measures

#### Clinical and Demographic assessment

1) Sociodemographic data will be obtained based by using an adapted form of the CIDI sociodemographic section;

2) Structured Clinical Interview for DSM-IV (SCID) I: SCID is a semi structured interview for the DSM-IV [71,72]. It allows the diagnosis of mental health disorders according to DSM IV criteria and has already been validated for Brazilian population [73];

3) Clinician Administered PTSD Scale (CAPS) [74]: A clinician rating scale for assessing current and lifetime PTSD: the CAPS-1. CAPS is a structured clinical interview designed to be applied by clinician and its validation was included as part of the first phase of this protocol. It is a 30 items scale investigating the frequency and intensity of PTSD symptoms and traumatic life experiences.

4) Beck Anxiety Inventory (BAI): BAI is a self-administered 21 items questionnaire assessing intensity of anxiety symptoms [75];

5) Beck Depression Inventory (BDI) is used to assess depressive symptoms in clinical settings [108]; it is a self-administered 21 items questionnaire, and it has been validated for the Brazilian population [76];

6) Global Assessment of Function (GAF) scale provides data on the clinical global state of patients [77];

7) The Social Adjustment Scale (SAS) is a self-administered instrument to assess social adaptation [78,79] and it has been validated to the Brazilian social and cultural context [80];

8) Medical Outcomes Study 36-Item Short-Form Health Survey (MOS SF-36) [81] is a self-report scale constructed to collect data on health status, functioning, and well-being. A Portuguese version of the questionnaire has already been tested for its validity and reliability in Brazil [82];

9) Early Trauma Inventory (ETI) is a semi-structured interview comprising 56 items to measure traumatic life experiences occurred in early life, in the following domains: sexual, physical and psychological abuse and other traumatic life experiences [83];

10) Clinical global Impressions (CGI) is a scale to assess treatment response in patients with mental disorders [84];

11) Peritraumatic Dissociative Experiences Questionnaire (PDEQ) [85] is a reliable and valid measure of peritraumatic dissociation as previously described in the epidemiologic study section.

#### The Neuropsychological Assessment

The neuropsychological evaluation will be performed in a single session by neurophysiologists trained in the instruments listed as follows. The training has been conducted by a senior psychologist, acquainted to the assessments chosen for the study, who will be responsible for supervising the trainees in order to keep the accuracy of measurements. The following tests will be part of the Neuropsychological Assessment:

1) The Wisconsin Card Sorting Test (WCST) will be used to assess cognitive set shifting and executive functions [86];

2) Vocabulary and Blocks – Subscales WAIS III is a widely used measure for intellectual level[87];

3) The Digit Span – Subscale WAIS III is an important tool for evaluating working memory and short-term memory[87];

4) The Spatial Span – Subscale WMS III is meant for assessment of immediate nonverbal memory and nonverbal working memory[87];

5) The International Affective Picture System (IAPS) was validated in Brazil and measures visual memory and emotional reaction through positive, negative and neutral figures [87];

6) The Rey Auditory-Verbal Learning Test (RAVLT) assesses the verbal learning and memory [88]

7) The Stroop Test is designed to assess selective attention and cognitive flexibility[89]

8) The Visual Reproduction – Subscale WMS III assess visual memory[87];

9) Cancellation of Mensulan assesses selective visual attention, vigilance and visual neglect[90];

10) Social and Occupational Functioning Assessment Scale (SOFAS) [1] – Assess the social and occupational functioning in the community.

#### Magnetic Resonance Imaging

Imaging data will be acquired at the Instituto do Sono, Federal University of Sao Paulo, using a GE1.5-T Signa scanner. Structural MR images will be acquired using a sagittal T1 acquisition series (TR = 9.8 ms, TE = 3.1 ms, flip angle = 30°, NEX = 1, matrix size = 256 × 256, FOV = 24 cm, thickness = 1.0 mm). A T2-weighted image series will also be acquired. Before scanning, a sagittal scout series (nine to eleven 5-mm-thick slices with a 1-mm interslice gap) will be performed to determine image quality and clarity as well as subject head position. Measurements will be conducted on PC workstation with the aid of BRAINS2 software [91]. Before tracing, T1- and T2-weighted images will be spatially realigned so that the brain anterior-posterior axis is parallel to the intercommissural line, which was horizontal in the sagittal plane, and the interhemispheric fissure is vertical in the axial plane. Six brain-limiting points (anterior, posterior, superior, inferior, left, and right) will then be picked to place images into the standard Talairach three-dimensional space [92]. After coregistering and fitting the three image sequences, a multimodal tissue classification will be performed using a Bayesian classifier based on discriminant analysis. This segmentation method automatically generates thresholds permitting the discrimination of grey and white matter as well as cerebrospinal fluid.

#### Hippocampus

The hippocampus will be traced manually on the coronal plane as described by Pantel et al [93]. Tracings begin with the generation of auxiliary guideline traces on the sagittal plane. The auxiliary traces are necessary to provide a neuroanatomically correct separation of rostral and caudal parts of the hippocampus from adjacent nonhippocampal brain tissue. Tracing will begin on the most medial slices. The starting slice is identified by choosing the slices that (going from medial to lateral) first show the cerebral peduncle separated from the upper pons. Once the anterior border of the hippocampus is identified on the starting slice, the vertical crosshairs will be placed anteriorly to this border. This procedure facilitates the identification of the anterior border on the following slices, because the head of the hippocampus, in general, does not extend beyond this level on the more lateral slices. The anterior border is outlined by the alveus and the uncal recess, which may be obliterated. Dorsally, CSF of the temporal horn of the lateral ventricle outlines the body, whereas the pulvinar thalamus serves as the border for the tail. On the medial slices the body is bordered by the fimbria, which is excluded from the trace itself. The posterior border is formed by the CSF of the lateral ventricle. The ventral border is defined by the WM of the temporal lobe.

#### Orbitofrontal cortex

The orbitofrontal cortex (OFC) will be outlined according to the proposed geometrical method developed by Lacerda et al [49]. The OFC will be manually measured in the coronal plane. The tip of the genu of corpus callosum will be located in the sagittal plane and used as the most posterior slice to be traced in the coronal plane. The last slice traced will be the most anterior coronal slice where brain tissue can be identified. The superior limit will be divided in two parts to reflect more the actual anatomical boundary of the OFC. In the subgenual regions, and specifically from the tip of the genu to the most anterior part of the CC, the superior boundary will be represented by the inferior border of the anterior cingulate corresponding to a midpoint at the interhemispheric fissure about five slices (5.08 mm) below the intercommissural line. More anteriorly, and specifically in the slices ahead of the genu of the CC, the superior limit will be represented by a midpoint placed on the intercommissural line. This "lowering" of the superior limit will be done to avoid inclusion of subgenual structures in the first slices that are not traditionally considered to be part of OFC (e.g., anterior cingulate).

In all slices, horizontal and vertical crosshairs will be placed as tangent lines at the inferior and lateral surfaces of the frontal lobes, respectively. The intersection of these two lines (horizontal and vertical crosshairs) will generate two lateral points that will be connected to the superior limit point, composing the lateral boundaries of the tracings. The inferior border will be traced following the inferior surface of the frontal lobes between the two lateral boundaries described above. The OFC will also be subdivided into gyrus rectus and orbital gyri by tracing a line through the olfactory sulcus. This subdivision will not be conducted in the most anterior slices where the olfactory sulcus disappears.

#### SPECT scans of Dopamine Transporter

The kits of TRODAT-1 were obtained through a scientific collaboration with the Research Institute of Nuclear Energy, Lung-Tan, Taiwan. The metastable technetium-99 was produced by a generator of [99Mo] (molybdenum-99) from the Institute of Nuclear Energy Research (IPEN-SP) with freshly elution. The kits of TRODAT-1 were marked with [99mTc] according to the technique developed by Kung et al[94]. Sixty mCi of elution of Sodium Pertechnetate [99mTc] diluted in 5 ml of saline solution are injected in the kit and submitted to 16 atmospheres and temperature of 120°C, during 30 minutes in an autoclave. Later, the solution of [99mTc] TRODAT-1 is cooled at room temperature.

The images will be acquired through a single photon emission computerized tomography (SPECT) Gama camera of the type (Hawkeye General Electric Medical System, USA) according to the methodology previously validated [95]. All subjects will receive an intravenous injection of 2 ml containing among 22 to 25 mCi of [99mTc]-TRODAT-1 in an antecubital peripheral vein. Images will be acquired 4 hours after the injection. The SPECT modality of the system with two heads and fan-beam collimators of ultra-high resolution will be used. Energy Window will be 140 ± 14 keV and matrix of 128 × 128 in circular orbit with step and shoot movements of 64 steps for each head will be used, with diameter and degree of rotation of 30 cm and 360°, respectively. The time of acquisition for the projection will be of 20 seconds, with a factor of zoom of 1.45. The reconstruction of the SPECT images will be accomplished through a filter algorithm of filtered retroprojection and a Butterworth filter of 0.4 cut off with pixels of 10th order. Three-dimensional images of the whole brain will be obtained and, for the analysis, two transaxial slices will be used at the level of the striatal body, with 3 mm of thickness corresponding to the level of the largest captation of the radiotracer. DAT density will be calculated with binding potential (DAT-BP) using regions of interests (ROI) bilaterally drawn in the striatum (STR) and the occipital cortex (OCC-background). BP will be calculated with the formula striatum (STR-OCC)/OCC.

#### Data management and Analyses

1) Questionnaires will be double typed and data will be entered on SPSS software data files. Participants will be divided into two categories, according to the diagnosis: (1) lifetime diagnosis of PTSD and (2) Traumatic experience, but no lifetime diagnosis of PTSD. The following comparisons will be carried out in the two groups:

1) Neuropsychological Study: neuropsychological measures of PTSD cases compared to controls;

2) Magnetic resonance imaging study: to assess neurostructural abnormalities in OFC and hippocampus of patients with PTSD compared to resilient controls;

3) Molecular neuroimaging study: dopamine transporter density using single photon emission tomography of PTSD cases compared to controls.

Data will be codified and analyzed using the Statistical Package for Social Sciences (SPSS for Windows, version 15.0). Proportion differences will be compared using the Chi Square test or Fisher's exact test, as appropriate. Continuous variables will be compared by Analysis of Variance (ANOVA) or Mann-Whitney test for non-parametric data. All significant tests will be considered as 2-tailed. P values < 0.05 will be considered statistically significant. Because of the exploratory nature of the study, we do not consider alpha adjustment in multiple comparisons and we did not calculate the sample size (Table 3). The main analyses will examine the relationship between exposure to traumatic events and the PTSD occurrence. Unadjusted relative risk for studied variables cognition, cortisol level, hippocampal volume, and dopamine transporter density (and 95% confidence intervals) are presented for trauma without PTSD and trauma with PTSD, both with and without adjustment for age (18–29 years, 30–39 years, 40–49 years, 50–60) and gender. As a secondary analysis, we will be adjusting for clinical and demographic variables previously associated with PTSD: marital status, country of birth, socio-economic status, urbanicity and employment status.

**Table 3:** Sample size in each study for PTSD cases and control groups

	Neuropsychological study		Structural Neuroimaging study		Molecular Neuroimaging study	
	
	Case	Control	Case	Control	Case	Control
Number of individuals for each group	100	100	60	60	25	25

PTSD = Posttraumatic stress disorder; MRI = Magnetic Resonance Imaging

#### Ethical Issues

Participants will be informed about research procedures and risks and signed an informed consent submitted and approved by the Ethical Committee of the Federal University of São Paulo (Processes: 1-Neuropsychology-0124/06; Neurostructural-1026/06; Molecular neuroimaging-0295/06). Subjects diagnosed as having any mental health disorder will be offered a referral to the out-patient clinic at the Federal University of Sao Paulo.

## Discussion

This study protocol illustrates a collaborative work of potential value to researchers interested in innovative and realistic investigation aimed at understanding the neurobiology of PTSD in a population-representative sample. This article describes the methodological responses to challenges in conducting translational research, where patients are identified from a real-world scenario in a population-based study and go through neuropsychological, neurostructural and molecular neuroimaging techniques.

A recent review of the literature found over 60 studies examining memory and attention performance in PTSD subjects, but few studies have examined learning, executive functioning, and emotional reaction in this population. A key aim of the present neuropsychological study is not only to assess those less explored domains but, also, try to clarify some discrepancies reported in literature by examining a more homogeneous, adequately controlled sample. Some inconsistencies in neuropsychological findings may be attributed at least in part to sample limitations. Neuropsychological deficits involving attention and memory have been replicated in different samples including war veterans [14,15,20], rape victims, and other traumatized populations. However, most neuropsychological studies of PTSD involve war veterans with chronic PTSD who frequently exhibit comorbid psychiatric conditions such as depression, anxiety and substance use disorders, which represent major confounders [15]. The effects of depressed mood on neuropsychological functioning have been well documented [96]. The high rate of comorbidity and overlap of symptoms between these two disorders, however, makes it difficult to exclude individuals with current depression from PTSD studies. Therefore, it is important to address the presence of comorbid depression and to include measures of depression as covariates in analyses.

Despite the unquestionable progress in identification of neurocognitve and neuroanatomical abnormalities associated with PTSD over the past decade or so, it remains unclear whether neurostructural and neuropsychological alterations are specific to PTSD or are related to unspecific environmental factors such as stress and substance abuse. The findings from this neuropsychological study, together with data from neurostructural investigation, may offer an uncommon opportunity to examine the convergence of cognitive and neuroanatomical alterations in patients with PTSD. Stress-induced functional and structural alterations in hippocampus and OFC may mediate many of the symptoms of PTSD that are related to memory dysregulation and hyperexcitability. Interestingly, reversion of both neuropsychological and neuroanatomical abnormalities has been demonstrated after treatment with paroxetine, which in turn has been shown to promote neurogenesis in animal studies [97]. The increasing use of sophisticated neuroimaging techniques is certainly enhancing our understanding of PTSD, potentially improving prevention, treatment, and cognitive rehabilitation programs [23].

Although a previous study showed involvement of DAT gene in PTSD [67], and other indirect investigations have suggested a connection between dopaminergic system and stress [61], insufficient research has been done on the role of the DAT in relation to PTSD. To the best of our knowledge this will be the first SPECT study investigating dopamine transporter density in patients with PTSD and well matched resilient controls coming from an epidemiologic sample. The results of this study will help to disentangle whether possible dopaminergic changes in PTSD are a "state condition" or a "trait" marker of this disorder, raising a discussion why some subjects exposed to trauma develop PTSD and some others do not. Moreover, further studies evaluating patients with PTSD, resilient controls and healthy control subjects (who never experienced trauma) would provide useful information to clarify whether dopaminergic abnormalities are related to PTSD itself or to psychological trauma exposure.

Advances in molecular imaging techniques, such as SPECT, have made important contributions to the understanding of the pathophysiology of neuropsychiatric disorders [98]. Molecular imaging approaches are more sensitive than Neuroanatomical imaging techniques, and are able to identify subtle cerebral pathophysiological changes before neurostructural abnormalities take place. One of the major goals of molecular imaging research has been the identification of biomarkers, which are defined as the characteristics that are objectively measured and can differentiate normal biologic processes from pathogenic processes. These approaches has the potential to provide accurate and early neuropsychiatric recognition, evaluate disease progression, and monitor treatment efficacy [68].

These studies have several important limitations. First, cases are recruited after they have developed PTSD. Assuming we find differences between cases and controls on neuropsychological/imaging variables, we will not be able to determine whether these differences reflect a risk factor or a consequence of the disorder. Second, in several situations both cases and controls, were exposed to traumatic experience years before the investigations, producing a potential recall bias.

This study protocol intends to be helpful for researchers and clinicians interested in designing and/or conducting translational research in the field of trauma and posttraumatic stress disorder.

## Competing interests

The authors declare that they have no competing interests.

## Authors' contributions

RAB, JJM, SBA, MFM, MRJ, WR, MIQ, IF have made a substantial contribution to the conception and design of the study and will be supervising data analysis and interpretation of data. ALTL and APJ participated in the structural neuroimaging planning of the project. RAB and MCS designed the molecular imaging section of the protocol. AFS, CA, JR, MH, and TSM are post-grad students involved in different parts of the project. CG and GB made a substantial contribution to the conception and design of the study, will be supervising data analysis and interpretation of data, particularly in the genoma analysis. DRL will be participating in the analysis and interpretation of data. JPF and LCQ are post-doc students and will be participating of data analysis and interpretation of the results. KCK will be supervising data analysis and interpretation of results. MCS did develop the SPECT study and is involved in data collection, analysis and interpretation of dopamine carriers. SM is a senior psychologist responsible for the neuropsychological assessments of the study (choice of instruments, training and accuracy of measurements).

## Pre-publication history

The pre-publication history for this paper can be accessed here:


